# Caloxanthone C: a pyran­oxanthone from the stem bark of *Calophyllum soulattri*

**DOI:** 10.1107/S1600536811036294

**Published:** 2011-09-14

**Authors:** Gwendoline Cheng Lian Ee, Siau Hui Mah, Huey Chong Kwong, Soek Sin Teh, Mohamed Ibrahim Mohamed Tahir, Sidik Silong

**Affiliations:** a Department of Chemistry, Faculty of Science, Universiti Putra Malaysia, 43400 UPM Serdang, Selangor, Malaysia

## Abstract

The title compound [systematic name: 5,10-di­hy­droxy-2,2-di­methyl-12-(2-methyl­but-3-en-2-yl)­pyrano[3,2-*b*]xanthen-6(2*H*)-one], C_23_H_22_O_5_, isolated from the stem bark of *Calophyllum soulattri*, consists of four six-membered rings and a 2-methyl­but-3-en-2-yl side chain. The tricyclic xanthone ring system is almost planar [maximum deviation = 0.093 (2) Å], whereas the pyran­oid ring is in a distorted boat conformation. The 2-methyl­but-3-en-2-yl side chain is in a synperiplanar conformation. There are two intra­molecular O—H⋯O hydrogen bonds. In the crystal, mol­ecules are linked by C—H⋯O inter­actions, forming a zigzag chain propagating in [010].

## Related literature

For related structures, see: Ee *et al.* (2010 ▶); Fun *et al.* (2006 ▶); Doriguetto *et al.* (2001 ▶); Boonnak *et al.* (2007 ▶); Ndjakou *et al.* (2007 ▶). For the biological activity of *Calophyllum* species, see: Dharmaratne *et al.* (1999 ▶, 2009 ▶); Zou *et al.* (2005 ▶); Ito *et al.* (1999 ▶, 2002 ▶); Ee *et al.* (2004 ▶). For standard bond lengths, see Allen *et al.* (1987 ▶).
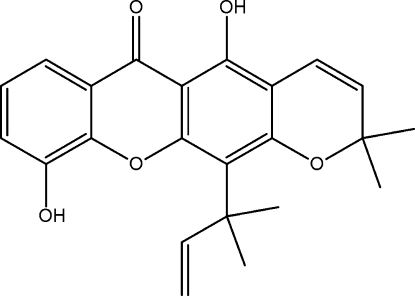


## Experimental

### 

#### Crystal data

C_23_H_22_O_5_
*M* *_r_* = 378.42
Monoclinic, 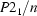

*a* = 6.7013 (3) Å
*b* = 15.8951 (7) Å
*c* = 17.3891 (7) Å
β = 93.181 (4)°
*V* = 1849.39 (14) Å^3^
*Z* = 4
Cu *K*α radiation
μ = 0.78 mm^−1^
*T* = 150 K
0.34 × 0.15 × 0.07 mm

#### Data collection

Oxford Diffraction Gemini diffractometer
Absorption correction: multi-scan (*CrysAlis PRO*; Oxford Diffraction, 2006 ▶) *T*_min_ = 0.890, *T*_max_ = 0.947
10133 measured reflections
3503 independent reflections
3048 reflections with *I* > 2σ(*I*)
*R*_int_ = 0.023

#### Refinement

*R*[*F* ^2^ > 2σ(*F* ^2^)] = 0.056
*wR*(*F* ^2^) = 0.170
*S* = 1.00
3488 reflections
254 parameters
H-atom parameters constrained
Δρ_max_ = 0.34 e Å^−3^
Δρ_min_ = −0.34 e Å^−3^

### 

Data collection: *CrysAlis CCD* (Oxford Diffraction, 2006 ▶); cell refinement: *CrysAlis RED* (Oxford Diffraction, 2006 ▶); data reduction: *CrysAlis RED*; program(s) used to solve structure: *SIR92* (Altomare *et al.*, 1994 ▶); program(s) used to refine structure: *CRYSTALS* (Betteridge *et al.*, 2003 ▶); molecular graphics: *Mercury* (Macrae *et al.*, 2006 ▶); software used to prepare material for publication: *CRYSTALS*.

## Supplementary Material

Crystal structure: contains datablock(s) I. DOI: 10.1107/S1600536811036294/su2302sup1.cif

Structure factors: contains datablock(s) I. DOI: 10.1107/S1600536811036294/su2302Isup2.hkl

Supplementary material file. DOI: 10.1107/S1600536811036294/su2302Isup3.cml

Additional supplementary materials:  crystallographic information; 3D view; checkCIF report

## Figures and Tables

**Table 1:** Hydrogen-bond geometry (Å, °)

*D*—H⋯*A*	*D*—H	H⋯*A*	*D*⋯*A*	*D*—H⋯*A*
O16—H16⋯O5	0.83	1.79	2.570 (2)	155
O28—H28⋯O1	0.81	2.25	2.690 (2)	115
C12—H12⋯O5^i^	0.94	2.51	3.441 (3)	168

Symmetry code: (i) 
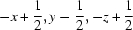
.

**Fig. 1.**
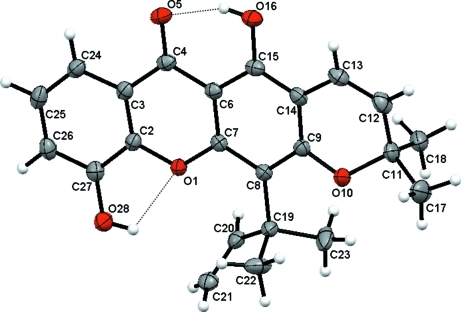
The molecular structure of the title molecule, with the crystallographic numbering scheme and displacement ellipsoids drawn at the 50% probability level. The intramoleular O-H···O hydrogen bonds are shown as dashed lines [see Table 1 for details].

**Fig. 2.**
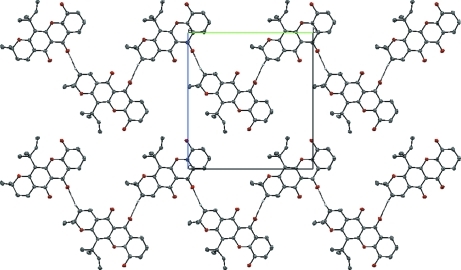
A view of the along the a-axis of the C-H···O hydrogen bonded (thin grey lines; see Table 1 for details) zizgzag chain in the crystal of the title compound [b-axis green; c-axis blue].

## supplementary crystallographic information

### Comment

*Calophyllum* species are native to tropical areas, mainly in Asia, Australia, Africa and Polynesia. This genus is well known for various bioactivities due to the existence of a variety of secondary metabolites such as xanthones (Dharmaratne *et al.*, 1999), coumarins (Ee *et al.*, 2004) and flavanoids (Ito *et al.*, 1999). Xanthones are known to have various biological activities such as, antifungal (Dharmaratne *et al.*, 1999), anti-oxidant (Dharmaratne *et al.*, 2009), anti-inflammatory (Zou *et al.*, 2005) and anti-cancer (Ito *et al.*, 2002). We present here the crystal structure of Caloxanthone C, isolated from the stem bark of *Calophyllum soulattri*.

The molecular structure of the title compound is illustrated in Fig. 1. The bond distances are in the normal range (Allen *et al.*, 1987) and together with the bond angles are comparable to those reported for other pyranoxanthone structures (Ee *et al.* 2010; Fun *et al.* 2006; Doriguetto *et al.* 2001), and other closely related structures (Boonnak *et al.*, 2007; Ndjakou *et al.* 2007).

The title molecule has a xanthone skeleton, which is essentially planar [maximum deviation 0.093 (2) Å for atom C14] with two intramolecular O-H···O hydrogen bonds (Fig. 1 & Table 1). Rings A (C2,C3,C24-C27), B (O1,C2-C4,C6,C7) and C (C6-C9,C14,C15) are practically coplanar, including atoms O28, O5, and O16, that are linked to them; the latter deviate from the individual mean planes by 0.009 (2) Å, 0.016 (2) Å, and 0.056 (2) Å, for O28 from ring A, O5 from ring B and O16 from ring C, respectively. Rings A and B nearly lie in the same plane, as they form a dihedral angle of only 0.46 (9)°, while rings B and C are inclined to one another by 4.25 (9)°. The mean planes of rings A and C, which intersect on a line approximately through the middle of ring B, are inclined to one another by 4.62 (10)°. The same dihedral angle is 7.78 (9) ° in the trihydroxy derivative of the title compound, reported on by (Fun *et al.*, 2006), and 7.75 (7) ° for a similar pyranoxanthone structure (12-Acetyl-6-hydroxy-3,3,9,9-tetramethylfuro[3,4-*b*]pyrano[3,2-*h*]xanthene-7,11(3*H*,9*H*)-dione ) reported on by (Ee *et al.*, 2010).

The mean torsional angle of ring D (C9,O10,C11-C14) is 21.08 (13)° and it adopts a conformation half way between an envelope and a half boat. This conformation is probably caused by the constraint of the C12═C13 double bond which results in considerable pucking of ring D, happening at C11. This conformation is similar to that observed in other pyranoxanthone structures, such as 12-Acetyl-6-hydroxy-3,3,9,9-tetramethylfuro[3,4-*b*]pyrano[3,2-*h*]xanthene-7,11(3*H*,9*H*)-dione (Ee *et al.*, 2010) and 12-(1,1-Dimethyl-2-propenyl)-5,9,10-trihydroxy-2,2-dimethyl-2*H*,6*H*-pyrano[3,2-*b*]xanthen-6-one (Fun *et al.*, 2006).

The orientation of the 2-methylbut-3-en-2-yl (C19—C23) side chain with respect to the benzene ring C is indicated by the torsion angle of C7—C8—C19—C20 = 27.6 (3)° [compared to 28.8 (3) ° in (Fun *et al.*, 2006)], indicating a synperiplanar conformation.

In the crystal, there is an intermolecular C—H···O hydrogen bond (Table 1, Fig. 2) the leads to the fomation of a zigzag chain propagating in [010].

### Experimental

The stem bark of *Calophyllum soulattri* was collected from the Sri Aman district in Sarawak, Malaysia. Approximately 1 kg of air-dried stem bark of *Calophyllum soulattri* was ground into a fine powder and extracted successively in a Soxhlet apparatus with n-hexane, dichloromethane, ethyl acetate and methanol for 72 h. The extracts were evaporated to dryness under vacuum to give 15.3 g of dichloromethane extract, which was subjected to column chromatography, over silica gel, several times. Stepwise gradient systems using n-hexane, dichloromethane, ethyl acetate and methanol and eluting through the columns resulted in separation and purification of the extract. Caloxanthone C, a yellowish crystal with the melting point of 210–212 °C was isolated. Single crystals, suitable for X-ray diffraction analysis, were prepared by the slow evaporation and diffusion of diethyl ether into a solution of Caloxanthone C in chloroform at room temperature.

### Refinement

The H atoms could all be located in a difference Fourier map. They were initially refined with soft restraints on the bond lengths and angles to regularize their geometry [O—H = 0.82 Å, C—H = 0.93 - 0.98 Å], after which the positions were refined with riding constraints, with *U*_iso_(H) = k × U_eq_(O,C), with k = 1.5 for OH and CH_3_ H-atoms and k = 1.2 for all other H-atoms.

### Crystal data

**Table:**

C_23_H_22_O_5_	*F*(000) = 800
*M**_r_* = 378.42	*D*_x_ = 1.359 Mg m^−^^3^
Monoclinic, *P*2_1_/*n*	Melting point: 189 K
Hall symbol: -P 2yn	Cu *K*α radiation, λ = 1.54184 Å
*a* = 6.7013 (3) Å	Cell parameters from 4840 reflections
*b* = 15.8951 (7) Å	θ = 71–4°
*c* = 17.3891 (7) Å	µ = 0.78 mm^−^^1^
β = 93.181 (4)°	*T* = 150 K
*V* = 1849.39 (14) Å^3^	Plate, yellow
*Z* = 4	0.34 × 0.15 × 0.07 mm

### Data collection

**Table:**

Oxford Diffraction Gemini diffractometer	3503 independent reflections
Radiation source: sealed x-ray tube	3048 reflections with *I* > 2σ(*I*)
graphite	*R*_int_ = 0.023
ω/2θ scans	θ_max_ = 70.9°, θ_min_ = 3.8°
Absorption correction: multi-scan (*CrysAlis PRO*; Oxford Diffraction, 2006)	*h* = −8→8
*T*_min_ = 0.890, *T*_max_ = 0.947	*k* = 0→19
10133 measured reflections	*l* = 0→21

### Refinement

**Table:**

Refinement on *F*^2^	Hydrogen site location: inferred from neighbouring sites
Least-squares matrix: full	H-atom parameters constrained
*R*[*F*^2^ > 2σ(*F*^2^)] = 0.056	Method = Modified Sheldrick *w* = 1/[σ^2^(*F*^2^) + ( 0.08*P*)^2^ + 2.61*P*] , where *P* = (max(*F*_o_^2^,0) + 2*F*_c_^2^)/3
*wR*(*F*^2^) = 0.170	(Δ/σ)_max_ = 0.0002304
*S* = 1.00	Δρ_max_ = 0.34 e Å^−^^3^
3488 reflections	Δρ_min_ = −0.34 e Å^−^^3^
254 parameters	Extinction correction: Larson (1970), Equation 22
0 restraints	Extinction coefficient: 27 (7)
Primary atom site location: structure-invariant direct methods	

### Special details

**Table:**

Geometry. Bond distances, angles etc. have been calculated using the rounded fractional coordinates. All su's are estimated from the variances of the (full) variance-covariance matrix. The cell esds are taken into account in the estimation of distances, angles and torsion angles
Refinement. For this compound, 10133 numbers of reflections were collected and measured during the refinement. Symmetry related reflections were measured more than once and after merging the symmetry equivalent reflections there were only 3503 reflection left. 15 more reflections were filtered, as σ cutoff was set as -3 and (sin*θ*/*x*)set to>0.01 (to eliminate reflection measured near the vicinity of beam stop) therefore numbers of reflection reduced to 3488.

### Fractional atomic coordinates and isotropic or equivalent isotropic displacement parameters (Å^2^)

**Table:**

	*x*	*y*	*z*	*U*_iso_*/*U*_eq_	
O1	0.2643 (2)	0.41026 (9)	0.57160 (8)	0.0229 (4)	
O5	0.2129 (3)	0.53596 (10)	0.36618 (9)	0.0307 (5)	
O10	0.2854 (2)	0.14802 (10)	0.44806 (9)	0.0278 (5)	
O16	0.2066 (3)	0.39365 (10)	0.29598 (8)	0.0303 (5)	
O28	0.3025 (3)	0.48977 (10)	0.70816 (9)	0.0353 (6)	
C2	0.2663 (3)	0.49629 (13)	0.56988 (12)	0.0210 (6)	
C3	0.2503 (3)	0.54285 (13)	0.50222 (12)	0.0218 (6)	
C4	0.2296 (3)	0.49750 (14)	0.42909 (12)	0.0230 (6)	
C6	0.2301 (3)	0.40664 (13)	0.43370 (12)	0.0203 (6)	
C7	0.2456 (3)	0.36432 (13)	0.50527 (11)	0.0197 (6)	
C8	0.2438 (3)	0.27680 (13)	0.51359 (12)	0.0208 (6)	
C9	0.2496 (3)	0.23212 (13)	0.44407 (12)	0.0214 (6)	
C11	0.2127 (4)	0.09367 (15)	0.38384 (13)	0.0309 (7)	
C12	0.2517 (4)	0.13517 (16)	0.30843 (13)	0.0317 (7)	
C13	0.2589 (3)	0.21808 (15)	0.30307 (13)	0.0272 (7)	
C14	0.2370 (3)	0.27046 (14)	0.37081 (12)	0.0226 (6)	
C15	0.2218 (3)	0.35722 (14)	0.36604 (12)	0.0230 (6)	
C17	0.3296 (5)	0.01284 (17)	0.39606 (16)	0.0449 (9)	
C18	−0.0109 (4)	0.07989 (16)	0.39128 (14)	0.0362 (8)	
C19	0.2517 (3)	0.23222 (13)	0.59319 (12)	0.0247 (6)	
C20	0.1635 (3)	0.28569 (13)	0.65545 (12)	0.0242 (6)	
C21	0.2403 (4)	0.29604 (14)	0.72643 (13)	0.0275 (7)	
C22	0.4673 (4)	0.20684 (17)	0.61492 (13)	0.0380 (8)	
C23	0.1135 (5)	0.15364 (17)	0.59174 (15)	0.0455 (9)	
C24	0.2559 (3)	0.63098 (14)	0.50689 (13)	0.0261 (7)	
C25	0.2754 (3)	0.66952 (14)	0.57769 (15)	0.0289 (7)	
C26	0.2914 (3)	0.62217 (15)	0.64509 (14)	0.0285 (7)	
C27	0.2857 (3)	0.53529 (14)	0.64196 (13)	0.0253 (6)	
H12	0.26450	0.10080	0.26490	0.0384*	
H13	0.28030	0.24340	0.25580	0.0331*	
H16	0.19700	0.44460	0.30570	0.0463*	
H171	0.31860	−0.00790	0.44700	0.0664*	
H172	0.28140	−0.02920	0.35990	0.0668*	
H173	0.46950	0.02260	0.38840	0.0665*	
H181	−0.03090	0.05220	0.43970	0.0529*	
H182	−0.07820	0.13340	0.38910	0.0531*	
H183	−0.06030	0.04520	0.35010	0.0534*	
H20	0.03660	0.31080	0.64190	0.0285*	
H211	0.36950	0.27610	0.74130	0.0327*	
H212	0.16730	0.32320	0.76280	0.0333*	
H221	0.55190	0.25610	0.62300	0.0565*	
H222	0.51750	0.17280	0.57450	0.0567*	
H223	0.47390	0.17320	0.66120	0.0565*	
H231	0.09990	0.13450	0.64360	0.0671*	
H232	−0.01540	0.17000	0.56910	0.0678*	
H233	0.16600	0.10830	0.56210	0.0677*	
H24	0.24530	0.66350	0.46210	0.0304*	
H25	0.27720	0.72990	0.58170	0.0349*	
H26	0.30550	0.64830	0.69310	0.0342*	
H28	0.29350	0.44000	0.69800	0.0529*	

### Atomic displacement parameters (Å^2^)

**Table:**

	*U*^11^	*U*^22^	*U*^33^	*U*^12^	*U*^13^	*U*^23^
O1	0.0319 (8)	0.0176 (8)	0.0189 (7)	−0.0017 (6)	−0.0004 (6)	−0.0005 (6)
O5	0.0401 (10)	0.0266 (9)	0.0250 (8)	0.0005 (7)	−0.0005 (7)	0.0077 (6)
O10	0.0396 (9)	0.0191 (8)	0.0238 (8)	0.0034 (7)	−0.0053 (7)	−0.0037 (6)
O16	0.0421 (10)	0.0300 (9)	0.0184 (8)	0.0007 (7)	−0.0007 (7)	0.0032 (6)
O28	0.0587 (12)	0.0246 (9)	0.0222 (8)	−0.0023 (8)	−0.0006 (7)	−0.0024 (6)
C2	0.0197 (10)	0.0174 (10)	0.0260 (11)	−0.0002 (8)	0.0012 (8)	0.0000 (8)
C3	0.0156 (10)	0.0217 (11)	0.0279 (11)	−0.0009 (8)	0.0006 (8)	0.0012 (8)
C4	0.0198 (10)	0.0244 (11)	0.0247 (11)	0.0003 (8)	0.0011 (8)	0.0041 (9)
C6	0.0163 (10)	0.0230 (11)	0.0214 (10)	−0.0002 (8)	−0.0003 (8)	0.0011 (8)
C7	0.0189 (10)	0.0212 (11)	0.0189 (10)	−0.0009 (8)	0.0001 (8)	−0.0015 (8)
C8	0.0215 (10)	0.0205 (11)	0.0200 (10)	−0.0011 (8)	−0.0019 (8)	0.0004 (8)
C9	0.0203 (10)	0.0197 (11)	0.0239 (11)	−0.0005 (8)	−0.0021 (8)	−0.0006 (8)
C11	0.0449 (14)	0.0216 (11)	0.0254 (11)	0.0011 (10)	−0.0049 (10)	−0.0080 (9)
C12	0.0371 (13)	0.0327 (13)	0.0250 (11)	0.0040 (10)	0.0001 (9)	−0.0102 (10)
C13	0.0272 (11)	0.0340 (13)	0.0203 (10)	0.0016 (9)	−0.0002 (8)	−0.0033 (9)
C14	0.0196 (10)	0.0265 (11)	0.0212 (10)	0.0008 (8)	−0.0019 (8)	−0.0026 (9)
C15	0.0211 (10)	0.0285 (12)	0.0191 (10)	0.0005 (8)	−0.0006 (8)	0.0032 (8)
C17	0.0671 (19)	0.0267 (13)	0.0397 (15)	0.0126 (13)	−0.0079 (13)	−0.0097 (11)
C18	0.0505 (16)	0.0276 (12)	0.0295 (12)	−0.0087 (11)	−0.0068 (11)	−0.0020 (10)
C19	0.0356 (12)	0.0187 (10)	0.0196 (10)	−0.0026 (9)	−0.0012 (9)	0.0013 (8)
C20	0.0268 (11)	0.0203 (10)	0.0255 (11)	−0.0029 (9)	0.0024 (8)	0.0049 (8)
C21	0.0334 (12)	0.0270 (11)	0.0225 (11)	−0.0013 (9)	0.0052 (9)	0.0015 (9)
C22	0.0502 (16)	0.0403 (14)	0.0233 (12)	0.0203 (12)	0.0004 (11)	0.0061 (10)
C23	0.079 (2)	0.0295 (14)	0.0283 (13)	−0.0245 (14)	0.0062 (13)	0.0016 (10)
C24	0.0226 (11)	0.0215 (11)	0.0339 (12)	0.0000 (8)	−0.0002 (9)	0.0055 (9)
C25	0.0225 (11)	0.0189 (11)	0.0452 (14)	−0.0012 (8)	0.0020 (10)	−0.0024 (10)
C26	0.0276 (12)	0.0253 (12)	0.0323 (12)	0.0001 (9)	−0.0001 (9)	−0.0085 (9)
C27	0.0248 (11)	0.0241 (11)	0.0270 (11)	−0.0021 (9)	0.0011 (9)	−0.0019 (9)

### Geometric parameters (Å, °)

**Table:**

O1—C2	1.368 (3)	C19—C20	1.522 (3)
O1—C7	1.365 (2)	C19—C22	1.527 (3)
O5—C4	1.253 (3)	C20—C21	1.321 (3)
O10—C9	1.359 (3)	C24—C25	1.375 (3)
O10—C11	1.473 (3)	C25—C26	1.392 (3)
O16—C15	1.348 (3)	C26—C27	1.383 (3)
O28—C27	1.359 (3)	C12—H12	0.9400
O16—H16	0.8300	C13—H13	0.9300
O28—H28	0.8100	C17—H171	0.9500
C2—C27	1.398 (3)	C17—H172	0.9600
C2—C3	1.389 (3)	C17—H173	0.9700
C3—C24	1.404 (3)	C18—H181	0.9700
C3—C4	1.462 (3)	C18—H182	0.9600
C4—C6	1.446 (3)	C18—H183	0.9500
C6—C7	1.413 (3)	C20—H20	0.9600
C6—C15	1.413 (3)	C21—H211	0.9400
C7—C8	1.399 (3)	C21—H212	0.9300
C8—C9	1.405 (3)	C22—H221	0.9700
C8—C19	1.553 (3)	C22—H222	0.9600
C9—C14	1.411 (3)	C22—H223	0.9700
C11—C17	1.514 (4)	C23—H231	0.9600
C11—C18	1.527 (4)	C23—H232	0.9600
C11—C12	1.504 (3)	C23—H233	0.9600
C12—C13	1.322 (3)	C24—H24	0.9300
C13—C14	1.457 (3)	C25—H25	0.9600
C14—C15	1.385 (3)	C26—H26	0.9300
C19—C23	1.554 (4)		
			
C2—O1—C7	121.12 (16)	C3—C24—C25	119.8 (2)
C9—O10—C11	119.21 (17)	C24—C25—C26	120.8 (2)
C15—O16—H16	104.00	C25—C26—C27	120.4 (2)
C27—O28—H28	109.00	C2—C27—C26	118.6 (2)
O1—C2—C27	115.10 (18)	O28—C27—C2	121.51 (19)
O1—C2—C3	123.42 (18)	O28—C27—C26	119.9 (2)
C3—C2—C27	121.48 (19)	C11—C12—H12	118.00
C4—C3—C24	122.92 (19)	C13—C12—H12	121.00
C2—C3—C24	118.83 (19)	C12—C13—H13	120.00
C2—C3—C4	118.25 (19)	C14—C13—H13	120.00
O5—C4—C3	121.2 (2)	C11—C17—H171	111.00
O5—C4—C6	122.38 (19)	C11—C17—H172	110.00
C3—C4—C6	116.38 (18)	C11—C17—H173	110.00
C4—C6—C7	121.59 (19)	H171—C17—H172	109.00
C7—C6—C15	117.78 (19)	H171—C17—H173	108.00
C4—C6—C15	120.60 (19)	H172—C17—H173	108.00
O1—C7—C8	116.46 (17)	C11—C18—H181	109.00
O1—C7—C6	119.23 (18)	C11—C18—H182	109.00
C6—C7—C8	124.32 (19)	C11—C18—H183	109.00
C7—C8—C19	123.08 (18)	H181—C18—H182	110.00
C7—C8—C9	114.40 (18)	H181—C18—H183	109.00
C9—C8—C19	122.35 (18)	H182—C18—H183	110.00
C8—C9—C14	123.77 (19)	C19—C20—H20	116.00
O10—C9—C8	117.82 (18)	C21—C20—H20	118.00
O10—C9—C14	118.20 (18)	C20—C21—H211	121.00
O10—C11—C17	104.15 (19)	C20—C21—H212	120.00
O10—C11—C12	109.73 (19)	H211—C21—H212	119.00
C12—C11—C18	110.8 (2)	C19—C22—H221	111.00
O10—C11—C18	107.74 (18)	C19—C22—H222	109.00
C12—C11—C17	112.4 (2)	C19—C22—H223	111.00
C17—C11—C18	111.7 (2)	H221—C22—H222	109.00
C11—C12—C13	120.5 (2)	H221—C22—H223	109.00
C12—C13—C14	120.5 (2)	H222—C22—H223	107.00
C9—C14—C13	118.7 (2)	C19—C23—H231	109.00
C9—C14—C15	119.00 (19)	C19—C23—H232	108.00
C13—C14—C15	122.1 (2)	C19—C23—H233	112.00
O16—C15—C6	120.75 (19)	H231—C23—H232	110.00
C6—C15—C14	120.28 (19)	H231—C23—H233	109.00
O16—C15—C14	118.92 (19)	H232—C23—H233	109.00
C8—C19—C22	109.03 (17)	C3—C24—H24	120.00
C8—C19—C23	111.18 (18)	C25—C24—H24	120.00
C20—C19—C23	101.88 (18)	C24—C25—H25	121.00
C22—C19—C23	110.3 (2)	C26—C25—H25	119.00
C20—C19—C22	111.78 (17)	C25—C26—H26	121.00
C8—C19—C20	112.58 (17)	C27—C26—H26	119.00
C19—C20—C21	126.0 (2)		
			
C7—O1—C2—C3	−0.1 (3)	O1—C7—C8—C9	−172.41 (17)
C7—O1—C2—C27	179.82 (17)	O1—C7—C8—C19	3.0 (3)
C2—O1—C7—C6	0.6 (3)	C6—C7—C8—C9	7.4 (3)
C2—O1—C7—C8	−179.54 (17)	C6—C7—C8—C19	−177.21 (19)
C11—O10—C9—C8	154.62 (19)	C7—C8—C9—O10	168.09 (17)
C11—O10—C9—C14	−30.5 (3)	C7—C8—C9—C14	−6.5 (3)
C9—O10—C11—C12	42.9 (3)	C19—C8—C9—O10	−7.3 (3)
C9—O10—C11—C17	163.42 (19)	C19—C8—C9—C14	178.13 (19)
C9—O10—C11—C18	−77.9 (2)	C7—C8—C19—C20	27.6 (3)
O1—C2—C3—C4	0.2 (3)	C7—C8—C19—C22	−97.0 (2)
O1—C2—C3—C24	−179.41 (18)	C7—C8—C19—C23	141.2 (2)
C27—C2—C3—C4	−179.74 (19)	C9—C8—C19—C20	−157.36 (19)
C27—C2—C3—C24	0.6 (3)	C9—C8—C19—C22	78.0 (2)
O1—C2—C27—O28	0.4 (3)	C9—C8—C19—C23	−43.8 (3)
O1—C2—C27—C26	179.23 (18)	O10—C9—C14—C13	1.2 (3)
C3—C2—C27—O28	−179.6 (2)	O10—C9—C14—C15	−173.23 (18)
C3—C2—C27—C26	−0.8 (3)	C8—C9—C14—C13	175.72 (19)
C2—C3—C4—O5	179.4 (2)	C8—C9—C14—C15	1.3 (3)
C2—C3—C4—C6	−0.8 (3)	O10—C11—C12—C13	−28.3 (3)
C24—C3—C4—O5	−1.0 (3)	C17—C11—C12—C13	−143.7 (2)
C24—C3—C4—C6	178.85 (19)	C18—C11—C12—C13	90.6 (3)
C2—C3—C24—C25	−0.5 (3)	C11—C12—C13—C14	2.4 (4)
C4—C3—C24—C25	179.91 (19)	C12—C13—C14—C9	13.0 (3)
O5—C4—C6—C7	−178.9 (2)	C12—C13—C14—C15	−172.8 (2)
O5—C4—C6—C15	3.3 (3)	C9—C14—C15—O16	−178.96 (19)
C3—C4—C6—C7	1.3 (3)	C9—C14—C15—C6	3.5 (3)
C3—C4—C6—C15	−176.56 (18)	C13—C14—C15—O16	6.8 (3)
C4—C6—C7—O1	−1.2 (3)	C13—C14—C15—C6	−170.74 (19)
C4—C6—C7—C8	178.96 (19)	C8—C19—C20—C21	−136.6 (2)
C15—C6—C7—O1	176.65 (18)	C22—C19—C20—C21	−13.5 (3)
C15—C6—C7—C8	−3.2 (3)	C23—C19—C20—C21	104.3 (3)
C4—C6—C15—O16	−2.2 (3)	C3—C24—C25—C26	0.5 (3)
C4—C6—C15—C14	175.28 (19)	C24—C25—C26—C27	−0.7 (3)
C7—C6—C15—O16	179.87 (19)	C25—C26—C27—O28	179.67 (19)
C7—C6—C15—C14	−2.6 (3)	C25—C26—C27—C2	0.8 (3)

### Hydrogen-bond geometry (Å, °)

**Table:**

*D*—H···*A*	*D*—H	H···*A*	*D*···*A*	*D*—H···*A*
O16—H16···O5	0.83	1.79	2.570 (2)	155
O28—H28···O1	0.81	2.25	2.690 (2)	115
C12—H12···O5^i^	0.94	2.51	3.441 (3)	168

Symmetry codes: (i) −*x*+1/2, *y*−1/2, −*z*+1/2.
